# Clinical validation of nursing diagnosis “00085 Impaired Physical Mobility” in multiple traumas victims*

**DOI:** 10.1590/1518-8345.2859.3190

**Published:** 2019-10-28

**Authors:** Raisa Camilo Ferreira, Erika Christiane Marocco Duran

**Affiliations:** 1 Universidade Estadual de Campinas, Faculdade de Enfermagem, Campinas, SP, Brazil.; 2 Centro Universitário de Itapira, Itapira, SP, Brazil.; 3 Scholarship holder at the Coordenação de Aperfeiçoamento de Pessoal de Nível Superior (CAPES), Brazil.

**Keywords:** Nursing Diagnosis, Validation Studies, Multiple Trauma, Patient Care Planning, Nursing Process, Decision Trees, Diagnóstico de Enfermagem, Estudos de Validação, Traumatismo Múltiplo, Planejamento de Assistência ao Paciente, Processo de Enfermagem, Árvores de Decisões, Diagnóstico de Enfermería, Estudios de Validación, Traumatismo Múltiple, Planificación de Atención al Paciente, Proceso de Enfermería, Árboles de Decisión

## Abstract

**Objective:**

to clinically validate the nursing diagnosis “Impaired Physical Mobility”, identifying its prevalence, defining characteristics, related factors, and associated conditions with the calculation of accuracy measures and generation of Decision Trees, as well as clinically and etiologically characterize the multiple traumas victims.

**Method:**

methodological, cross-sectional study of clinical validation type, using diagnostic accuracy measures and generating decision tree.

**Results:**

the sample consisted of 126 patients, 73% male, with a mean age of 38.29 years. The frequency of the nursing diagnosis studied was 88.10%; the defining characteristic with the highest prevalence was “Difficulty turning” (58.73%), with a predictive power of 98.6%; the associated condition “Alteration in bone structure integrity” stood out with 72.22%. The accuracy measures also indicated their predictive power.

**Conclusion:**

the components aforementioned were considered predictors of this diagnosis. This study contributed to improve the identification of clinical indicators associated with advanced methods of diagnostic validation, directing care and reducing the variability present in clinical situations.

## Abstract (pt)

**Objetivo:**

validar clinicamente o diagnóstico de enfermagem “Mobilidade Física Prejudicada”, identificando sua prevalência, características definidoras, fatores relacionados e condições associadas pelo cálculo das medidas de acurácia e geração de árvores de decisão, assim como caracterizar clínica e etiologicamente as vítimas de múltiplos traumas.

**Método:**

estudo metodológico, transversal, do tipo validação clínica com utilização das medidas de acurácia diagnóstica e geração de árvore de decisão.

**Resultados:**

a amostra foi composta de 126 pacientes, sendo 73% do sexo masculino, com média de idade de 38,29 anos. A frequência do diagnóstico de enfermagem estudado foi de 88,10%; a característica definidora com maior prevalência “Dificuldade para virar-se” (58,73%) teve um poder de predição de 98,6%; a condição associada “Alteração na integridade das estruturas ósseas” destacou-se com 72,22%. As medidas de acurácia também apontaram seu poder preditivo.

**Conclusão:**

foram considerados preditores desse diagnóstico os componentes citados acima. Este estudo contribuiu com o aprimoramento na identificação dos indicadores clínicos associados a métodos avançados de validação diagnóstica, direcionando a assistência e reduzindo a variabilidade presente nas situações clínicas.

## Abstract (es)

**Objetivo:**

validar clínicamente el diagnóstico de enfermería “Deterioro de la movilidad física”, identificando su prevalencia, las características definitorias, los factores relacionados y las condiciones asociadas mediante el cálculo de las medidas de exactitud y de la elaboración de Árboles de Decisión, así como caracterizar clínica y etiológicamente las víctimas de múltiples traumas.

**Método:**

estudio metodológico, transversal, del tipo validación clínica con la utilización de medidas de exactitud diagnóstica y elaboración de árbol de decisión.

**Resultados:**

la muestra constó de 126 pacientes, el 73% del sexo masculino, con una media de edad de 38,29 años. La frecuencia del diagnóstico de enfermería en estudio fue del 88,10%; la característica definitoria con mayor prevalencia “Dificultad para girarse” (58,73%) presentó una predicción del 98,6%; la condición asociada “Pérdida de la integridad de las estructuras óseas” se destacó con el 72,22%. Las medidas de exactitud también apuntaron su poder predictivo.

**Conclusión:**

se consideraron predictores de ese diagnóstico los componentes citados. El presente estudio contribuyó a mejorar la identificación de los indicadores clínicos asociados a métodos avanzados de validación diagnóstica, orientando la atención y reduciendo la variabilidad presente en las situaciones clínicas.

## Introduction

It is estimated that trauma is responsible, annually, for 5.8 million deaths worldwide and 125 thousand in Brazil. However, deaths are not the only problem. For each recorded death there are 13 hospital discharges and 140 visits to emergency services. Only in the state of São Paulo, in 2017, 153 thousand hospitalizations were registered^(^ 1 ^-^ 4 ^)^.

Research shows that in half of the traumatic occurrences, associated injuries occur-two or more serious injuries in at least two areas of the body-or multiple lesion-two or more serious injuries in one area of the body - characterizing multiple trauma, which represents a serious and complex health problem^(^ 1 ^-^ 2 ^)^.

To optimize the treatment, identify the areas of injury, and correctly estimate clinical severity, it is necessary to analyze the mechanism of the event and each lesion individually. For this, there are several severity score systems^(^ 5 ^)^. In the university hospital where this research project was carried out, the systems adopted are: Abbreviated Injury Scale (AIS), Injury Severity Score (ISS), Revised Trauma Score (RTS), and Trauma and Injury Severity Score (TRISS), described following.

The Abbreviated Injury Scale derives from the consensus of the Association for the Advancement of Automotive Medicine, which describes each type of injury and its score according to its relative severity per region of the body (head, face, thorax, abdomen, extremities, external injuries, or other traumatic injuries, including lacerations, bruises, abrasions and burns) on a 6-point scale, being 1 = minimum and 6 = maximum, related to intractable injuries^(^ 5 ^)^.

It constitutes the basis for the calculation of the Injury Severity Score, calculated by the sum of the squares of the three systems that scored the most in the AIS, ranging from 0 to 75; scores greater than 16 are considered severe. In addition, if an injury is assigned an AIS of 6 points (intractable injury), the ISS score is automatically assigned to 75^(^ 5 ^)^.

As for the Revised Trauma Score, it is calculated from the first information on the values of the Glasgow Coma Scale (GCS), Systolic Blood Pressure (SBP) and respiratory rate (RR), classified with scores ranging from 4 (normal) to 0 (severely compromised); through the equation RTS = 0.9368 GCS + 0.7326 SBP + 0.2908 RR. The result ranges from 0 to 7.8408 and is subsequently converted to percentages that indicate the probability of survival (PS), according to this example: 7.8408 (98.8%); 7 (96.9%); 6 (91.9%); 5 (80.7%); 4 (60.5%), 0-3 (2.7-0.1%)^(^ 5 ^)^.

The Trauma and Injury Severity Score also determines the probability of survival, however, it uses the equation PS = 1/(1+e^-b^), with *e* being the basis of the Napierian logarithm and *b* derived from the multiple regression analysis of the Major Trauma Outcome Study database, calculated using the values of ISS, RTS, age, and penetrating or blunt trauma^(^ 5 ^)^.

The complexity of these patients referred to hospitals for specialized treatment and uninterrupted care stands out, needing advanced technologies for life support, reduction of complications, treatment of injuries, and recovery^(^ 2 ^,^ 6 ^)^.

In this context of care, nurses are responsible for identifying problems, managing nursing care and employing systematic practices to guide care and increase its efficacy, quality and safety. Having as an ally the nursing process (NP), a methodological and scientific tool, it is a private and mandatory activity for nurses, which guides this care and determines the suitable treatment plan, consisting of five interdependent and inter-related steps^(^ 7 ^)^.

The second stage is the nursing diagnosis (ND) defined as a “clinical judgment on a human response to health conditions/life processes, or a vulnerability to such response, from an individual, family, group, or community.”^(^ 8 ^)^ It is classified by standardized systems such as taxonomy II of NANDA International, Inc (NANDA-I) adopted in this study. This standardized language system presents each ND along with its theoretical definition, defining characteristics (DC), consisting of observable or communicable signs and symptoms that substantiated their presence and related or risk factors (RF) and associated conditions (AC), which are not independently modifiable by the nurse, but contribute to the occurrence of ND^(^ 8 ^)^.

For the multiple trauma victims, the nursing diagnosis “00085 Impaired Physical Mobility”, instituted in the aforementioned taxonomy in the year of 1973 and updated in 2017, stands out. It belongs to domain 4 activity/rest, Class 2 activity/exercise, defined as “limitation in independent purposeful movement of the body or of one or more extremities”^(^ 8 ^)^ and it is composed by 14 defining characteristics, 18 related factors and 10 associated conditions^(^ 8 ^)^.

We emphasize that, although only the RF are susceptible to alteration by the nursing interventions, the AC favor the identification of this ND and relate directly to the clinical profile of the population, so that its accurate identification helps in the inferential process^(^ 8 ^)^.

They correlate with the study population, since the latter presents structural alterations and physiological imbalance in the organism, induced by the exchange of energy between tissues and the medium, during trauma, causing changes in mobility^(^ 1 ^-^ 2 ^)^. Researchers who evaluated the frequency of ND focusing on multiple traumas victims verified a prevalence between 42.3% and 81.0%^(^ 9 ^-^ 14 ^)^, judging it as characteristic of this population. We emphasize the need to develop diagnostic validation studies that may legitimize the aforementioned diagnosis in this population, directing care and incrementing the scientific quality of nursing care^(^ 14 ^)^.

The development of researches on diagnostic validation have been recently increasing for the review and evaluation in different populations of the constituent elements of nursing diagnoses, aiming to contribute to diagnostic accuracy, scientific substantiation, and refinement of the set of clinical indicators to direct the care provided. In this way, it allows its use by nurses in diverse populations, making them susceptible to generalization by their refinement, improvement, and theoretical-practical articulation, benefiting communication and nursing records^(^ 9 ^,^ 16 ^)^.

Validation studies aims to evaluate the representativeness and predictive power degree of the constituent elements of each ND by listing the set of characteristics present in the clinical environment, which determine the presence or absence of the diagnosis in a given context or scenario. Its conduction should occur with direct observation of the DC, RF, and AC of the investigated diagnosis, and their frequencies feed the statistical analyses^(^ 15 ^)^.

Thus, the objective was to clinically validate the nursing diagnosis “Impaired Physical Mobility,” identifying its prevalence, defining characteristics, related factors, and associated conditions by the calculation of accuracy measures and generation of Decision Trees, as well as to clinically and etiologically characterize the multiple traumas victims.

## Method

A methodological study, a clinical validation model, recommended by the literature as the third stage of the validation process and diagnostic accuracy^(^ 17 ^)^.

Data collection took place at the *Hospital das Clínicas* of the State University of Campinas, in the Intensive Care Units (ICU), Referenced Emergency Unit and nursing wards (Orthopedics, Traumatology, Neurosurgery, and Trauma Surgery), from August 2017 to January 2018. Adult patients with a medical diagnosis of multiple traumas were included according to the definition presented^(^ 4 ^)^. Patients with previous motor and sensory deficits^(^ 16 ^-^ 17 ^)^ were excluded.

The sample size calculation was based on the formula for finite populations, equivalent to 188 patients; the estimated proportion was 0.50. The sample error and significance level assumed were 5.0%. The final sample size was 126 patients^(^ 18 ^)^.

All nurses members of the “Study and Research Group on Nursing Care Management” participated in an 8-hour classroom training, conducted by the researchers, to discuss the inferential process and the topics related to the polytraumatized patients. Previous validation studies were discussed and the data collection instruments^(^ 15 ^-^ 16 ^)^ were presented.

On this occasion, they were also submitted to 12 clinical histories, applied in a single round, since the inferential diagnostic process is not considered something perfectly accurate. Therefore, the repetition of the application could present cognitive memory, influencing this process^(^ 15 ^,^ 19 ^-^ 21 ^)^. The clinical histories were elaborated by the researchers portrayed patients with multiple traumas, and in half of the cases the studied ND was present, and absent on the other half^(^ 15 ^-^ 16 ^)^.

The answers were evaluated concerning efficacy (ability of the diagnostician to correctly detect the presence or absence of ND); false negative rate (chance to classify ND as absent when present); false positive rate (chance to classify ND as present when absent), and tendency (predisposition of the diagnostician to accept or reject the ND, calculated by the ratio between false positive and false negative rates). These are classified into three cutoff points: acceptable, marginal, and unacceptable. Those who achieved acceptable levels of scoring, i.e., efficacy ≥ 0.9; false positive ≤ 0.05; false negative ≤ 0.02; and trend values in the range of 0.80-1.20 were considered apt^(^ 22 ^)^.

The collection was performed by three nurses (value determined by convenience, as there is no methodological recommendation), called diagnosticians, after approval during the training. In this type of research, the diagnostic inference performed by the diagnosticians represents the imperfect gold standard, considering that nursing diagnoses are human phenomena and there are no ideal reference standards, in addition to the absence of devices for objective measurement^(^ 15 ^-^ 16 ^)^.

Each patient was evaluated only once by a diagnostician, employing the instruments with the conceptual and operational definition of each defining characteristic of the IPM diagnostics^(^ 23 ^)^, in addition to the list of related factors and associated conditions, clinical data, sociodemographic characterization, origin, profession, religion, education level, trauma mechanism, severity score systems, International Classification of Diseases (ICD 10), Instrument for the NP^(^ 23 ^)^, and two copies of the informed consent form^(^ 15 ^-^ 16 ^)^.

The induction of the decision tree occurred by the database, containing the diagnostic prevalence as an outcome (dependent variable) and that of its components (independent variables). We opted to work with the algorithm Chi-square Automatic Interaction Detection (CHAID). As basic parameters for generation, we determined: significance level for division of nodes and categories fusion of 0.05, and likelihood ratio as a method to obtain the Chi-square value^(^ 24 ^)^.

The data were stored in spreadsheets of the Excel® software. The statistical analysis performed with the support of the Statistical Analysis System (SAS) software, version 9.4, and the Statistical Package for Social Science (SPSS), version 22.0 were: comparison of unpaired Student’s t test and Mann-Whitney’s test; of association for qualitative variables, Chi-square and Fisher’s exact test; and Poisson’s logistic regression for dichotomous variables^(^ 16 ^-^ 17 ^)^.

This study was approved by the Research Ethics Committee, under opinion number 1.947.516. Data collection occurred after explaining the study and signing of the informed consent form by the patients or their caretakers^(^ 17 ^)^.

## Results

The sample was comprised of 73.0% (n=92) male patients, with a mean age of 38.3 years; standard deviation (SD) of 12.9 years; median of 36 years; minimum 18 and maximum 60 years. Individuals without partners were 53.2% (n=67); 54.3% (n=44) declared themselves to be Catholics, 29.6% (n=24) practiced evangelical religions, and 16.5% (n=13) declared adhesion to other practices, among them Presbyterians, Seventh-day Adventists, Spiritism, and atheists.

Regarding education level, 52.7% (n=58) answered to have high school completed, 30.9% (n=34) finished elementary school, and 16.4% (n=18) completed higher education. The percentage that represented economically active population was 79.6% (n=90).

Table 1 presents the clinical characterization data of the patients, which subsidized the calculations of the adopted trauma scores.

**Table 1:** Clinical characterization of multiple traumas victims admitted to a teaching hospital in the countryside of the state of São Paulo (n=126). Campinas, SP, Brazil, 2017-2018

Variables	Mean	SD*	Median	Minimum	Maximum
Body Mass Index	27.50	4.94	26.8	20.00	45.70
Systolic Blood Pressure^†^	119.66	25.30	120.00	0.00	180.00
Diastolic Blood Pressure^†^	72.03	15.99	73.50	0.00	110.00
Noninvasive Mean Arterial Pressure	87.91	18.12	90.00	0.00	125.33
Heart Rate^†^	88.29	19.96	86.00	55.00	150.00
Respiratory Rate^†^	18.01	3.83	18.00	2.00	30.00
Glasgow Coma Scale	12.09	3.79	15.00	3.00	15.00

* SD – standard deviation;

† Systolic and Diastolic blood pressure values, Heart rate and Respiratory rate – were measured by the multiparameter monitors (pressure values marked as zero refer to cases in which they were not quantified by the monitor, i.e., the values were below 30 mmHg)

The polytraumatism etiology was heterogeneous, with emphasis on traffic accidents, with 62.0% (n=78). Of these, 70.7% (n=53) were caused by motorcycles, followed by falls 27.6% (n=22); melee weapon injuries 3.2% (n=4); interpersonal aggressions, gunshot wounds, and suicide attempted by hanging with 1.6% (n=2) each; crushing, burns, and foreign body ingestion, each presented 0.8% (n=1). We observed that 7.1% (n=9) of the patients were under the influence of alcohol at the time of the trauma. The traumas were classified as blunt (92.9%) and penetrating (7.1%).

The mean value observed for ISS was 25.73; SD 14.73; median of 21.50; minimum of six and maximum of 75, with approximately 50% of severe traumas. RTS equaled 7.23; SD 1.33; median 7.84; minimum of 0.29 and maximum of 7.84. In 84.80% (n=106) of the cases, the score was higher than seven, and, after conversion to percentage, it indicated a probability of survival above 91.9%. Only 1.60% (n=2) had scores lower than five and PS below 7%. TRISS mean was 84.17; SD 25.90; median 96.20; minimum of 0.70 and maximum of 99.5. Thus, indicating a probability of survival higher than 90.0% in 72.2% (n=91) of cases.

There were several ICD listed, with emphasis on T07 - Unspecified multiple traumas (48.4%); S72 - Femur fracture (9.5%); S82 - Leg fracture, including ankle (6.3%); S06 - Intracranial trauma (4.0%); and S52 - Forearm fracture (3.2%).

The organic systems affected by the traumas were: bones (92.1%); integumentary (65.9%); muscular (36.5%); nervous (34.9%); respiratory (27.0%); digestive (13.5%); urinary (11.9%); cardiovascular (9.5%); lymphatic (1.6%); and reproductive (0.8%). It is noteworthy that more than one item should be marked regarding this characteristic.

The patients commonly presented more than one device or intervention, with emphasis on peripheral venous catheter, present in 76.2% (n=96), central venous catheter in 22.2% (n=28), orthopedic devices in 63.50% (n=80), delayed bladder catheter in 46.0% (n=58), nasoenteral catheter in 20.6% (n=26), and drains in 15.9% (n=20). Regarding the interventions, 21.4% (n=27) underwent mechanical ventilation; 18.3% (n=23) sedation, and 53.2% (n=67) surgery.

It was observed that 46.1% (n=59) had some morbidity, and the most frequent were systemic arterial hypertension (31.0%), Diabetes mellitus (15.5%), dyslipidemia (5.2%), and alcohol abuse (3.4%).

Concerning origin, 38.9% (n=49) were from the metropolitan region of Campinas; 28.6% (n=36) were from Campinas; 17.4% (n=22) were from cities of the countryside region of the state of São Paulo; 11.1% (n=14) from other states; and 4.0% (n=5) from São Paulo.

The prevalence of ND IPM was 88.1% (n=111). When this was not present, we identified diagnoses of Acute pain (00132) (46.7%); Impaired comfort (00214), Acute confusion (00128), Decreased cardiac output (00029), and Impaired skin integrity (00046) (13.3%, each). In addition to the ND IPM, Acute pain (00132) (28.0%), Dysfunctional ventilatory weaning response (00034) (16.0%), Risk of peripheral neurovascular dysfunction (00086) (12.0%), Decreased cardiac output (00029), Impaired oral mucous membrane integrity (00045), and Ineffective breathing pattern (00032) (8.0%, each) were also identified; as well as Impaired comfort (00214), Readiness for enhanced coping (00158), Impaired skin integrity (00046), Disturbed sleep pattern (000198), and Risk for unstable blood glucose level (00179) (4.0%, each).

For statistical purposes, defining characteristics and factors related to over 10 observations were considered. With higher prevalence, the DC “Difficulty turning” (58.7%) and RF “Alteration in bone structure the integrity” (72.2%) were found, as described in Tables 2 and 3.

**Table 2:** Prevalence of defining characteristics and related factors of the Nursing Diagnosis “Impaired Physical Mobility” in multiple traumas victims admitted to a teaching hospital in the countryside of the state of São Paulo (n=126). Campinas, SP, Brazil, 2017-2018

Variables	Presence		Absence	
	n*	%^†^	n*	%^†^
Defining characteristics				
Difficulty turning	74	58.73	52	41.27
Discomfort	55	43.65	71	56.35
Decrease in gross motor skills	22	17.46	104	82.54
Decrease in range of motion	13	10.32	113	89.68
Alteration in gait	12	9.52	114	90.48
Exertion dyspnea	8	6.35	118	93.65
Decrease in fine motor skills	8	6.35	118	93.65
Slowed movement	7	5.56	119	94.44
Postural instability	2	1.59	124	98.41
Uncoordinated movement	2	1.59	124	98.41
Decrease in reaction time	2	1.59	124	98.41
**Related factors**				
Pain	7	5.56	119	94.44
Decrease in muscle control	3	2.38	123	97.62
Disuse	1	0.79	125	99.21
Activity intolerance	1	0.79	125	99.21
Decrease in muscle mass	1	0.79	125	99.21
Reluctance to initiate movement	1	0.79	125	99.21

* n = absolute value;

† % = percentage value

**Table 3:** Accuracy measures, defining characteristics, and related factors of the Nursing Diagnosis “Impaired Physical Mobility” in multiple traumas victims admitted to a teaching hospital in the countryside of the state of São Paulo (n=126). Campinas, SP, Brazil, 2017-2018

Variable	Se*	Sp^†^	PPV^‡^	NPV^§^	Prevalence	p-value
Defining characteristic						
Difficulty turning	0.6577	0.9333	0.9865	0.2692	65.72	< 0.0001‖
Discomfort	0.4685	0.8000	0.9455	0.1690	46.85	0.0491‖
Decrease in gross motor skills	0.1982	1.0000	1.0000	0.1442	19.82	0.0713¶
Decrease in the range of motion	0.1171	1.0000	1.0000	0.1327	11.71	0.3625¶
Alteration in gait	0.1081	1.0000	1.0000	0.1316	10.81	0.3575¶
**Related factors**						
Pain	0.0360	0.8000	0.5717	0.1008	3.60	**
Decrease in muscular control	0.0270	1.0000	1.0000	0.1220	2.70	**
Disuse	0.0090	1.0000	1.0000	0.1200	0.09	**
Activity intolerance	0.0090	1.0000	1.0000	0.1200	0.09	**
Decrease in muscle mass	0.0090	1.0000	1.0000	0.1200	0.09	**
Reluctance to initiate movement	0.0090	1.0000	1.0000	0.1200	0.09	**

* Se = sensitivity;

† SP = specificity;

‡ PPV = positive predictive value;

§ NPV = negative predictive value;

‖ p-value – obtained by Chi-square test;

¶ p-value – obtained by Fisher’s exact test;

** It was not possible to define the p-value for RF because the number of observations was small

The DC “Engages in substitutions for movement”, “Movement-induced tremor” and “Spastic movement,” as well as additional DC “Muscular hypertonia,” “Muscular hypotonia” and “Muscle stiffness” had a frequency of less than 3.0%, with no occurrences registered by the diagnosticians.

The RF “Anxiety”, “Body mass index (BMI) > 75^th^ percentile appropriate for age and gender”, Cultural belief regarding acceptable activity”, “Decrease in muscular strength”, “Depression”, “Insufficient environmental support”, “Insufficient knowledge of value of physical activity”, “Joint stiffness”, “Malnutrition”, “Physical deconditioning”, and “Sedentary lifestyle” were not recorded by the diagnosticians.

The AC “Alteration in metabolism”, “Developmental delay” and “Contractures” were also not identified.

Regarding the AC, they presented, respectively, concerning absolute values and percentage: Alteration in the bone structure integrity (n=91 and 72.22%); Pharmaceutical agent (n=20 and 15.87%); Prescribed movement restrictions (n=18 and 14.29%); Alteration in cognitive functioning (n=11 and 08.73%); Musculoskeletal impairment (n=07 and 05.56%); Sensory-perceptual impairment (n=05 and 03.97%); Neuromuscular impairment (n=02 and 01.59%).

The DC “difficulty turning” presented higher values of sensitivity, specificity, and positive predictive value with statistical significance, being considered as a predictor of this diagnosis. The other DC presented specificities and high positive predictive values and negative predictive values below the cutoff point.

The DC “Discomfort” obtained 53.2% of false negatives, i.e., they did not present the DC although presenting the ND in question. Similar behavior to DC “Alteration in gait,” “Decrease in gross motor skills,” and “Decrease in the range of motion,” thus indicating that the absence of these DC warns of the ND absence by its values of specificity and positive predictive value, as demonstrated by the accuracy measures described in Table 3 below.

We also warn, due to the low number of RF occurrence, that the data presented are not statistically significant, but are important to characterize the sample.

We emphasize that although the AC are not factors modifiable by nursing interventions, they were determinant for the correct identification of the presence or absence of ND IPM, besides characterizing the population as they reflect situations that contribute to the occurrence of the ND; therefore, they also had their diagnostic accuracy measures calculated, of which stood out: “Pharmaceutical agent,” “Alteration in cognitive function,” and “Prescribed movement restrictions,” whose absence assumes that of the ND, as shown in the following Table.

**Table 4:** Accuracy measures of the associated conditions of the Nursing Diagnosis “Impaired Physical Mobility” in victims of multiple traumas admitted to a teaching hospital in the countryside of the state of São Paulo (n=126). Campinas, SP, Brazil, 2017-2018

Variable	Se*	Sp^†^	PPV^‡^	NPV^§^	Prevalence	p-value‖
Associated conditions						
Alteration in bone structure integrity	0.7477	0.4667	0.9121	0.2000	74.77	0.1212
Pharmaceutical agent	0.1802	1.0000	1.0000	0.1415	18.02	0.1260
Prescribed movement restrictions	0.1532	0.9333	0.9444	0.1296	15.32	0.6938
Alteration in cognitive functioning	0.0991	1.0000	1.0000	0.1304	9.91	0.3583

* Se = Sensitivity;

† Sp = Specificity;

‡ PPV = positive predictive value;

§ NPV = negative predictive value;

‖ p-value obtained by Fisher’s exact test

Unpaired Student’s t test was performed for the variable age and the Mann-Whitney’s test for the variable days of hospitalization, both, without statistically significant results.

To verify the occurrence of the association between the qualitative variables and the diagnostic occurrence, the Chi-square test was performed, indicating its association with the variable marital status without a partner (p-value = 0.0067). There was also an association obtained by Fisher’s exact test with the variable TRISS<90 (p-value = 0.0362).

Poisson’s regression was used, one of the generalized linear models, to enable the interpretation of the relationship of the dependent variable “presenting the ND IPM,” with the independent variables (days of hospitalization, age, male, not having a partner, complete elementary school, undergoing surgeries during hospitalization, and ISS>24), risk factors or contributors brought by the literature for the occurrence of the traumatic event or alteration in mobility. A significant difference (p-value = 0.0121) was observed only between the variable marital status “without partner” and the presence of the ND in question (prevalence ratio = 0.82; confidence interval of 95% [0.70-0.96]).

**Figure 1:**
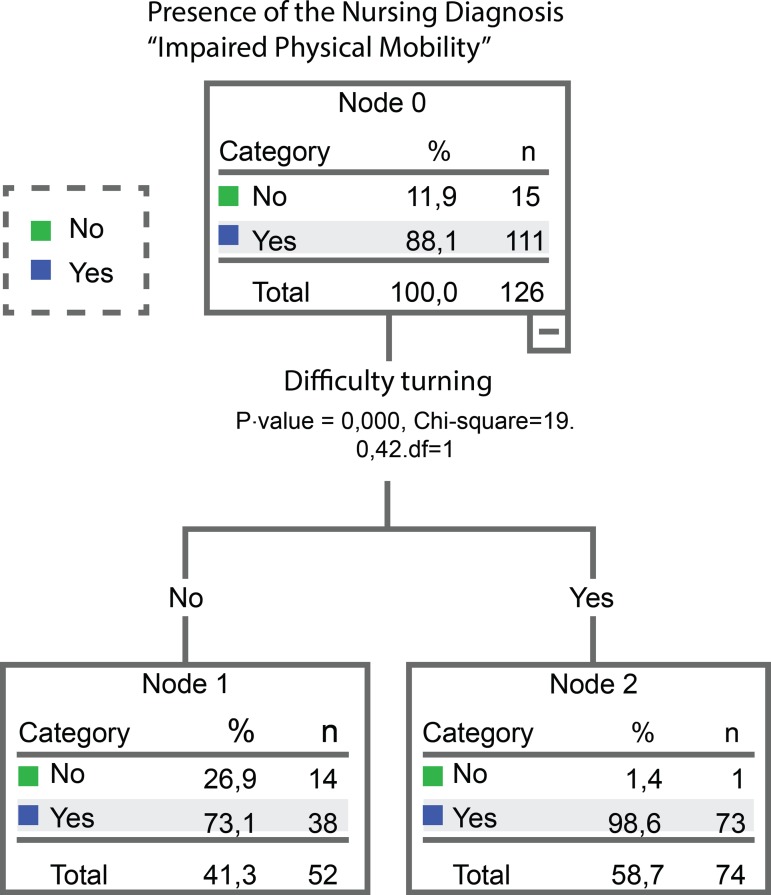
Decision tree generated with the predictive defining characteristic of the Nursing Diagnosis “Impaired Physical Mobility” in victims of multiple traumas, using the CHAID growth method (n=126). Campinas, SP, Brazil, 2017-2018

By multivariate analysis of the data, the induction of the decision tree was made, generated by the CHAID algorithm, with two nodes, being one a terminal node (Figure 1), offering conditional probabilities to the occurrence of the ND associated with the occurrence of DC to estimate the prediction of a set of data for the diagnosis, evidencing as a predictor “Difficulty turning”.

## Discussion

The prevalence of male was identified, agreeing with the literature^(^ 1 ^,^ 25 ^-^ 27 ^)^, which shows greater involvement of men aged between 20 and 40 years. The greatest vulnerability is associated with socio-cultural and economic factors, which can be related to individual behavior and personality, such as hyperactivity, impulsiveness and aggressiveness, lack of prudence and maturity in transit, non use of safety devices such as seat belt or helmet, driving under the influence of alcohol or drugs, performing more dangerous activities, and inattention^(^ 2 ^)^.

A religious transition was observed in the country, although it is still predominantly Catholic, followed by the evangelical religion. A growth in the non-Christian denominations was also noticed, as well as the number of people who declared to be atheists^(^ 28 ^)^.

We noticed an increase in the national education level^(^ 29 ^)^, reaching 11 years of study in the population aged 25 years or more, increasing from 33.6% to 42.5%, as found in the research project.

Approximately 80.0% of the sample was economically active, resulting in an important economic impact due to lost productive years, premature death, or years lived with disabilities. The expenditures with health care of this population are around U$300 billion/year and mean recovery time of 12 to 24 months^(^ 27 ^)^.

The main mechanism of trauma was the traffic accident, especially those caused by motorcycles, followed by the falls and injuries caused by weapons and aggression, predominantly affecting the musculoskeletal, integumentary, and nervous systems, supporting the findings of other studies^(^ 25 ^-^ 27 ^,^ 30 ^-^ 34 ^)^.

The literature^(^ 33 ^-^ 34 ^)^ justifies the greater involvement of extremities and head due to a greater vulnerability to injuries by direct trauma (fall or ejection from the vehicle) and because they are more unprotected, causing alterations, fractures, or dislocations in different places and portions, generating distinct ICD and, above all, unspecified multiple traumas.

Regarding the characterization of the clinical data, a research project^(^ 30 ^)^ that aimed to describe the clinical and sociodemographic profile of trauma victims from motorcycles, observed that 83.0% of the sample had GCS values equal to 15, differently from this study, which presented a mean value of 12.1.

It is noteworthy that, despite the greater clinical severity found (ISS>24) in 47.6% (n=60) of the traumas considered equal or higher than severe, the probability of survival indicated by RTS and TRISS was higher than 90%, reflecting values considered good^(^ 31 ^-^ 36 ^)^.

The victims that had ND IPM not identified showed better scores in the severity systems, demonstrating that individuals who did not present the ND had higher probability of survival and less severe traumas compared to those who presented it. Thus, a direct relationship between the occurrence of this ND and the severity of the trauma was observed^(^ 14 ^)^.

It is also noteworthy the occurrence of the surgical approach as the main form of treatment for correction or fixation of fractures, relief of intracranial pressure, drainage of hematomas, identification or correction of internal injuries, which, in association with the prevalence of blunt traumas, corroborate the findings^(^ 1 ^,^ 5 ^,^ 25 ^-^ 27 ^,^ 33 ^-^ 34 ^)^.

The presence of morbidities remained within the rates found, with emphasis on non-communicable chronic diseases, mainly associated with tobacco, sedentary behavior, alcohol abuse, and poor eating habits, which delay the recovery of these patients and or aggravate their clinical picture^(^ 35 ^)^.

The prevalence of the ND IPM was 88.1%. Other studies, with different objectives, showed lower prevalences^(^ 9 ^-^ 11 ^,^ 13 ^)^. It is noteworthy that the diagnosticians who performed the diagnostic inference, in this study, participated in processes that aimed to improve their inferential power, therefore, the results found reflected more precision to reality^(^ 15 ^)^.

The nursing diagnoses additional to the IPM are similar to the ones identified in the literature^(^ 14 ^)^, with emphasis on “Acute pain” and “Impaired skin integrity”. Above all, studies explaining the frequencies of these components are scarce, hindering a comparison of these findings.

Previously, during the content analysis, conceptual and operational definitions of DC were elaborated, which were submitted to evaluation by nurses specialized in NP and multiple traumas. The judgement reflected as the most prominent DC: “Decrease in gross motor skills” and “Difficulty turning,” as they presented a weighed mean ≥ 0.8, and less prominent, “Discomfort” (0.74), “Decrease in range of motion” (0.76), and “Alteration in gait” (0.75)^(^ 23 ^)^.

Thus, the content analysis step already pointed to the results identified in this clinical validation, which demonstrates the subsidy presented by analysis prior to the clinical validation^(^ 22 ^)^.

A prevalence of 72.2% (n=91) of AC “Alteration in bone structure in integrity” compatible with the predominant etiological origin was verified, in which there is an exchange of energy between the external environment and the human body, causing injuries throughout the organism with emphasis in orthopedic trauma (45.0 to 65.0%), comprising fractures, skin injuries, or muscle injuries resulting from traumas^(^ 1 ^,^ 33 ^)^.

Concerning accuracy, the DC “Difficulty turning” can be considered as a predictor of ND, with a prevalence of 65.72%. Conceptually defined by “requirement of a greater effort than usual to place oneself or move in a position different from that in which one was previously placed, and, operationally, signs (hesitation, ease of pain, use of objects, and support), verbal report of difficulty during the realization of the movement or inability to accomplish it,” thus explaining the limitations of this population^(^ 23 ^)^.

One of the factors contributing to the occurrence of this DC was the presence of two or more devices, which hinders the movement due to the fear of accidental removal, pain caused by manipulation, discomfort, and/or greater clinical severity, evidenced by the presence of respiratory support or sedation^(^ 1 ^,^ 33 ^)^. Other researchers also identified this DC as clinical evidence of the occurrence of the IPM diagnosis in the presence of devices^(^ 36 ^-^ 37 ^)^.

The DC “discomfort,” defined as “lack of feeling of well-being resulting from the state of physiological, physical and psychological harmony between the human being and the environment, which implies the presence of unpleasant sensations, pain, stress, restlessness, and operationally as a verbal report of discomfort, pain (ease of pain), stress, perceived lack of comfort, vague complaint of weakness, fatigue, and exhaustion”^(^ 23 ^)^, evidenced the characteristics of multiple traumas in the presence of devices.

A study that investigated the occurrence of this ND in hospitalized older adults considered the DC “Discomfort” in 36.7% of the older patients^(^ 38 ^)^, close to what was found (46.85%).

The DC “Alteration in gait,” understood as “involuntary changes in the set of movements, more or less rhythmic, of the lower limbs that promote the movement of the individual and, to evaluate it, it is necessary to analyze the gait, to verify the need for an aid device, the ability to change direction, evaluate if during the gait cycle the hip shifts only through two arches of movement during the stride,”^(^ 23 ^)^ had a prevalence of 10.81%. Other studies, with different populations, showed higher prevalences, of 28.4%^(^ 37 ^)^ and 86.7%^(^ 38 ^)^.

The DC “Decrease in gross motor skills” can be understood as “diminished ability to perform physical abilities involving muscular groups that give or receive strength from the objects, such as sitting down, using the upper limbs, running, bending down. The identification in the care practice is due to the assessment of the patient’s ability to mobilize large muscular groups that produce strength of the trunk, arms and legs, requesting them to sit and move arms and legs.”^(^ 23 ^)^ It was identified in 19.8% of the sample, inferior to the sample found in older adults victims of cerebrovascular accident (49.50%)^(^ 37 ^)^.

With prevalence of about 11% in this study, the DC “Decrease in range of motion,”, defined as “reduction of the natural distance and direction in which a joint moves, indicating the lack of mobility of one or more specific joints, noticed due to the ability of the body to perform small and large range movements, free of any restriction,”^(^ 23 ^)^ was present in 24.8% of the population in another study^(^ 37 ^)^.

We emphasize that the evaluation of DC “Alteration in gait,” “Decrease in gross motor skills,” and “Decrease in range of motion” in multiple traumas victims can be prevented by clinical severity and/or presence of orthopedic devices, which explains the lower occurrence^(^ 34 ^)^.

Approximately 74.8% of this population exhibited AC “Alteration in bone structure integrity”, understood by the “presence of normal state modification, which is not unharmed; continuity dissolution of one or more bones and/or displacement of one or more bones out of their normal position in the joint.”^(^ 1 ^)^ Only 8.4% who had this AC did not present the diagnosis, also considered a predictor of the IPM diagnosis by other researchers^(^ 9 ^,^ 14 ^)^.

The AC “Pharmaceutical agent,” defined as “use of medications that interfere with mobility due to its analgesic, sedative or other classes that alter the cognitive state,”^(^ 39 ^)^ characterize the medications widely used by these victims.

In turn, “Alteration in cognitive function,” understood as “any disturbance in the phases of the information process, such as perception, learning, memory, attention, vigilance, reasoning, problem solving, and psychomotor functioning (reaction time, movement time, speed of performance)”^(^ 40 ^)^, was present in 53.3% of the patients in the investigation that aimed to characterize hospitalized older adults and associate IPM with sex, age, presence of Diabetes mellitus, systemic arterial hypertension, pain, and physical activity^(^ 41 ^)^.

The AC “Prescribed movement restrictions,” described as “order or advice provided by the health team to limit the ability to move freely or to reach any other part of the body, or mechanical restraint for therapeutic and resting purposes”^(^ 42 ^)^, is associated with a high rate of musculoskeletal system affections, necessitating resting for recover^(^ 33 ^)^.

Although AC are optional components of these ND enunciation, they need to directly reflect the clinical situations, medical diagnosis, and other conditions that favor the occurrence of the ND^(^ 8 ^)^, a fact identified in this research project, which they perfectly reflected the clinical profile of this population, justifying the calculation of the accuracy measures.

The fact that some of the DC and AC present sensitivity values below the cutoff point, with high false negative values (absence of CD/RF/AC in the presence of the ND)is verified, which tenuously interfere in the accuracy of nursing care given to patients. Whereas, the presence of false positives (presence of DC/RF/AC in judgment in the absence of ND), interferes especially in the nursing care plan, since it neglects the identified clinical evidence, hindering the elaboration of results and nursing interventions^(^ 8 ^,^ 15 ^)^.

The statistical tests showed that not having a partner is related to the occurrence of the ND IPM, possibly because the age group affected by multiple traumas is predominantly composed by young people who are not yet in stable unions, being more influenced by the factors previously described^(^ 33 ^)^. As a protective factor for the occurrence of this ND, we have TRISS scores > 90, demonstrating lower severity^(^ 32 ^)^.

Scholars on nursing diagnoses affirm that to attest the validity of a given diagnosis it is necessary to submit it to clinical validation through advanced statistical methods, preferably comparing the findings between at least two different methods to increase their relevance and robustness, and to strengthen the clinical utility of the diagnostic indicators^(^ 8 ^,^ 15 ^)^.

In this case, when comparing the results obtained by the accuracy measurements to what was identified by the decision tree generation, it was verified, by both, the high predictive power of the DC “Difficulty turning,” present in 98.6% of the patients.

In the current edition of NANDA-I, we were confronted by the recategorization of many RF to AC (items that are not treatable independently by nurses); as it is something recent, it will require adaptation and adequacy regarding our clinical practice, since they are important and may aid the nurse when analyzing and confirming diagnoses, as demonstrated by the findings in this study. We also warn that we should not confuse them with DC and RF, components that may be intervened or altered by nurses.

Considering the aforementioned, the scarce number of studies about this ND for multiple traumas patients stands out, hindering the comparison of the findings, associated with the absence of research projects using two statistical methods for diagnostic accuracy.

The limitation of this study was the extension of the data collection time from four to five months.

## Conclusion

The objectives of this study were achieved and the diagnosis of “Impaired Physical Mobility” showed high prevalence among victims of multiple traumas, being considered characteristic of this population.

Clinical validation researches provides the identification of clinical evidences that lead to the accurate determination of nursing diagnoses, contributing to its refinement and stimulus for the use of the Nursing Process.

Although there are few studies addressing this identification in the aforementioned population, the advanced statistical methods aid the evaluation of data that enable us to ensure the reliability of the findings. In this context, decision trees and accuracy measures make the diagnostic inference process easier, improving their accuracy.

The occurrence of the defining characteristic “Difficulty turning” and/or of the associated condition “Alteration in bone structure integrity” was considered predictive of this diagnosis. The absence of the defining characteristics “Alteration in gait,” “Discomfort,” “Decrease in range of motion” and “Decrease in gross motor skills,” and of the associated conditions “Pharmaceutical agent,” “Alteration in cognitive functioning” and “Prescribed movement restrictions” are determinant in the identification of its non-occurrence.

It is noteworthy that, by correctly identifying the presence of the nursing diagnosis, it is possible to provide the appropriate nursing and therapy interventions to optimize the results, aiming to avoid clinical decline and permanent sequelae.
